# COVID-19 mortality and use of intensive care among ethnic minorities – a national register-based Danish population study

**DOI:** 10.1007/s10654-023-00991-9

**Published:** 2023-05-16

**Authors:** Marie Norredam, Sabrina Islamoska, Jørgen Holm Petersen, Thomas Benfield

**Affiliations:** 1 Danish Research Centre for Migration, Ethnicity and Health, Section of Health Services Research, Department of Public Health, University of Copenhagen, Øster Farimagsgade 5, Copenhagen, 1014 Denmark; 2 Section of Biostatistics, Department of Public Health, University of Copenhagen, Øster Farimagsgade 5, Copenhagen, 1014 Denmark; 3 Department of Infectious Diseases, Copenhagen University Hospital - Amager and Hvidovre, Hvidovre, Denmark; 4 Department of Clinical Medicine, Faculty of Health and Medical Sciences, University of Copenhagen, Copenhagen, Denmark

**Keywords:** COVID-19, Ethnic minorities, Mortality, Intensive care, Infections

## Abstract

Migrants and ethnic minorities are disproportionately affected by the Coronavirus Disease 2019 (COVID-19) pandemic compared to the majority population. Therefore, we studied mortality and use of mechanical ventilation (MV) by country of birth and migrant status in a nationwide cohort in Denmark. Nationwide register data on all cases hospitalized for > 24-hours with COVID-19 between February 2020 and March 2021. Main outcome measures were mortality and MV within 30 days of hospitalization for COVID-19. Odds ratios (OR) and 95% confidence intervals (95% CI) were estimated by region of origin and migrant status using logistic regression analyses, adjusting for age, sex, comorbidity and sociodemographic factors. Of 6,406 patients, 977 (15%) died and 342 (5%) were treated with mechanical ventilation. Immigrants (OR:0.55;95%CI: 0.44–0.70) and individuals of non-Western origin had a lower odds (OR: 0.49; 95% CI: 0.37–0.65) of death upon admission with COVID-19 compared to Danish born individuals. Immigrants and descendants (OR: 1.62; 95% CI: 1.22–2.15) as well as individuals of non-Western origin (OR: 1.83; 95% CI: 1.35–2.47) had a significantly higher odds of MV compared to Danish born individuals. Outcomes of individuals with Western origin did not differ. Immigrants and individuals of non-Western origin had a significantly lower COVID-19 associated mortality compared to individuals of Danish origin after adjustment for sociodemographic factors and comorbidity. In contrast, the odds of MV was higher for immigrants and individuals of non-Western origin compared to individuals of Danish origin.

## Introduction

Coronavirus Disease 2019 (COVID-19) has shown disturbing health inequalities worldwide asmigrants and ethnic minorities have been affected to a higher degree than the majority population. Across Europe and the United States, remarkably higher positive rates of Severe Acute Respiratory Syndrome Coronavirus-2 (SARS-CoV-2) and subsequent hospitalization with COVID-19 occur among migrant and ethnic minorities compared to majority populations [1–5]. In Europe, populations of non-Western origin from South East Asia and Africa appear to have increased risks of infection and hospitalization [1]. This overrepresentation of COVID-19 infections has mainly been explained by circumstances related to impaired access to care, comorbidity, low socioeconomic status as well as communication barriers and health literacy [1–6]. The role of all these different factors partly depended on national contexts.

In 2021, 10% and 4%, respectively, of the Danish population were either immigrants and descendants, of which 58% and 83% were of non-Western origin [7]. Further, immigrants and descendants of non-Western origin accounted for 25.7% of those who tested positive for SARS-CoV-2 while these groups represent 8.9% of the general population [8]. Similarly, the risk of hospitalisation due to COVID-19 infection was almost twice as high among all immigrants and descendants of non-Western origin – especially those from Somalia, Iraq, Pakistan, Morocco, and Lebanon compared to Danish-born individuals by Danish-born parents [9]. Almost half of this excess risk was explained after adjustment for age, sex, comorbidities and socioeconomic confounders such as income, education, frontline job exposure, number of household members and neighbourhood density.

Studies on disease severity and mortality from COVID-19 among migrants and ethnic minorities from the UK, Norway and United States have shown an alarming tendency to poorer outcomes compared to the majority population [10–12]. National data from the UK on use of intensive care showed that 34% of those hospitalised had BlackAsianMinorityEthnic (BAME) background although they account for 14% of the population [13]. In contrast, a recent meta-analysis of 58 studies from across the world found that although the risk of COVID-19 infection was higher in most ethnic minorities, once hospitalised, there was no clear inequality [14]. These conflicting findings have emphasized the need for large-scale data – preferably nationwide - on the risk of death and potential underlying causes of COVID-19 complications and death for migrants and ethnic minorities including the contribution of socioeconomic, demographic, and comorbidity risk factors [15]. Second, in the wake of the pandemic and possible future pandemics, studies are needed from other European immigration countries, which have different compositions of migrants and ethnic minorities and where other contextual factors are at play. Therefore, we aimed to study mortality and use of mechanical ventilation among ethnic minorities compared to ethnic Danes in a nationwide cohort of the Danish population.

## Methods

### Study population

**Fig. 1:**
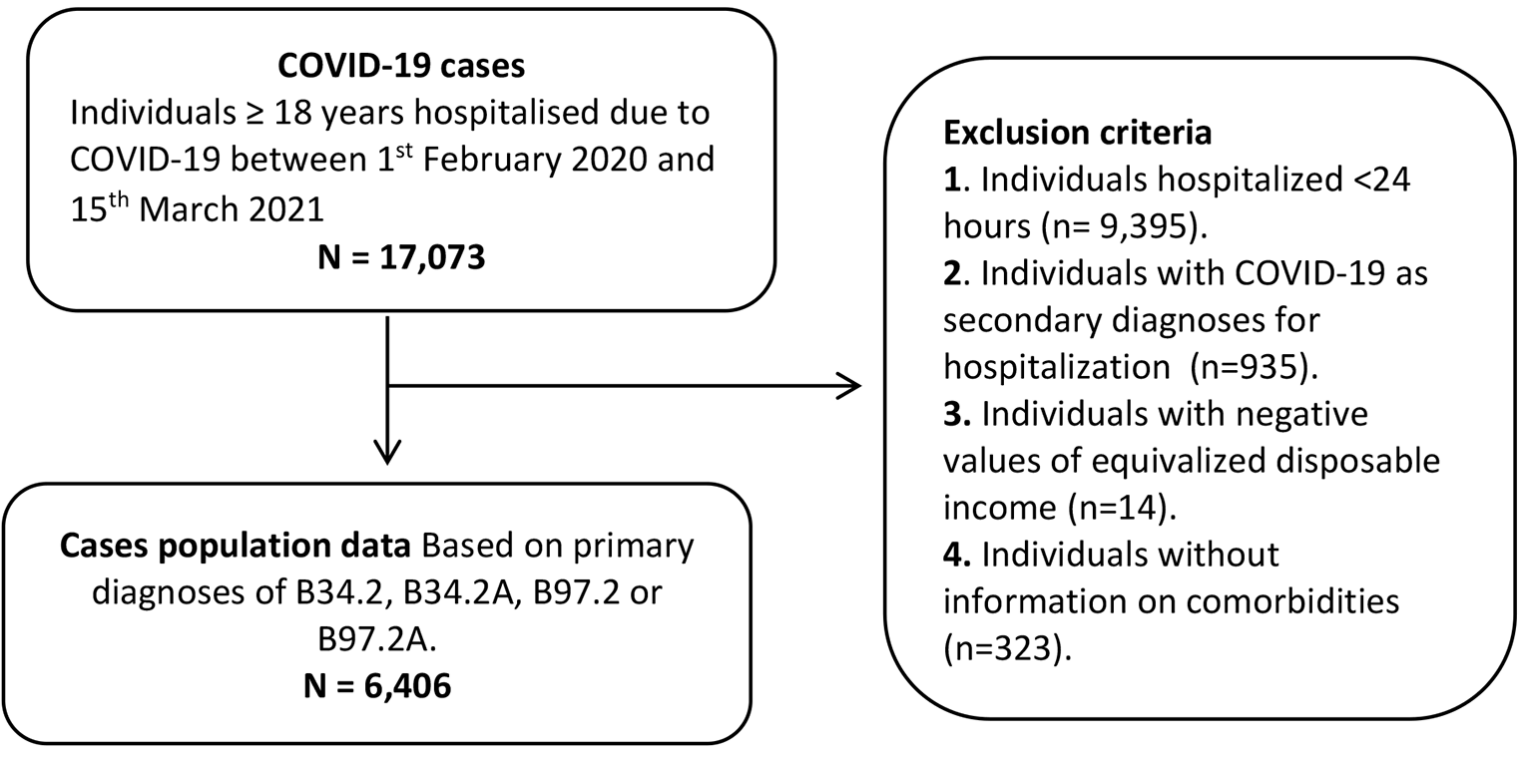
Selection of the study population

This nationwide register study was based on data of all individuals residing in Denmark aged 18 years or older on January 1st 2020. We included all patients admitted between February 1st 2020 and March 15th 2021 with a primary COVID-19 diagnosis according to ICD-10 (B34.2, B34.2 A, B97.2, or B97.2 A) as used previously [16]. National register-based hospital data on cases of COVID-19 were obtained from The National Patient Register, which includes information on diagnoses whenever an individual is in contact with any hospital department by inpatient, outpatient, or emergency room visits [17]. To increase the validity of COVID-19 diagnoses, COVID-19 cases were defined as inpatients hospitalized for ≥ 24 h and only with COVID-19 as the primary reason for hospitalization. Patients who were initially hospitalised for < 24 h, but readmitted within 7 days, were included. We excluded individuals who were (i) hospitalized with a secondary COVID-19 ICD-10 code; (ii) who had missing or negative income values or (iii) who lacked information on comorbidity (see Fig. 1). Vital statistics including in- and out-hospital death were obtained from the Civil Registration Registry [18].

## Variables

### Exposure

The exposure variables were ‘regional origin’ and immigrants or descendants (using mother’s country of birth) versus Danish origin. Information on country of birth was obtained from Statistics Denmark and categorization into non-Western versus Western was preformed according to their definitions [7]. We divided into three overall regional categories: (i) *Danish born* (a person of Danish origin who has at least one parent, who is a Danish citizen born in Denmark), (ii) *Western* (all EU countries, Andorra, Australia, Canada, Iceland, Liechtenstein, Monaco, New Zealand, Norway, San Marino, Switzerland, USA or the Vatican); and ii) *non-Western* (all other countries)^19^. We further divided into three geographical regions origin: (i) Asia; (ii) Africa and (iii) Europe, as these were the main world regional origins represented in our data. This was done according to World Bank regional definitions [19].

### Outcomes

Outcome variables were mortality and need of MV among COVID-19 hospitalised patients during follow-up from 1th of Februar 2020 until 1th of July 2021. Thirty-day mortality was calculated from the day of admission. Data on MV while receiving intensive care treatment was retrieved from the Danish Intensive Care Database.

### Covariates

Covariates included the following seven socioeconomic and demographic variables obtained from Statistics Denmark: (i) length of residence; (ii) marital status; (iii) highest-attained educational level (ISCED) [20]; (iv) persons per household: range: 1 to ≥ 5 persons; (v) occupation defined according to industry; (vi) equivalised disposable household income in Danish Kroner (DKK) divided into quartiles; and (vii) population density calculated by number of total individuals in a province divided by area in km^2^.

Further comorbidity was included as a covariate based on information on all ICD-10 diagnoses from The National Patient Register registered between 1st January 1980 and 31st December2020.^21^ Comorbidities were weighted by the Charlson Comorbidity Index (CCI) to create a continuous weighted comorbidity score [21].The comorbidity scores assigns weights from 1 to 6 to each comorbidity for every individual, which then receives a sum of weights based on all present comorbidities in an individual. A comorbidity score of 0 represents no comorbidities; higher scores indicate more severe comorbidities. The CCI was categorized into a score of 0, 1, 2, or ≥ 3.

## Statistical analyses

In descriptive analyses, the distribution of comorbidity, socioeconomic and demographic factors was investigated among individuals of Danish, Western and non-Western origin, who were hospitalised with COVID-19, died with COVID-19, and had been treated with MV. Logistic regression models were used for the main analyses. First, the odds ratios (OR) of death with 95% confidence intervals (95% CI) was calculated among immigrants and descendants, individuals of Western origin and non-Western origin, and individuals originated from Africa, Asia, or Europe. Second, the risk of mechanical ventilation was investigated for immigrants/descendants, individuals of Western and non-Western origin, and individuals from Africa, Asia, or Europe. Third, the risk of death was also examined among patients receiving mechanical ventilation. Individuals of Danish origin were used as the reference group for all analyses. Six step-wise models were applied to adjust for sex, age, CCI, household size, education and income.

For all analyses, SAS Enterprise Guide version 7.1 was used with a statistical significance level of 0.05. The study is approved by the University of Copenhagen Ethics Board, reference number 514 − 0231/18-3000. Individual consent is not required by Danish legislation for register-based studies.

## Results

Of 17,073 individuals admitted with a diagnosis of COVID-19, 9,395 had been admitted for less than 24 h, 935 individuals had COVID-19 as a secondary diagnosis for hospital contact, 323 had no information on comorbidity and 14 had missing information or negative income (see Fig. 1). Thus, a total of 6,406 individuals had a primary hospital admission for COVID-19.

Table 1 describes population characteristics. Among hospitalised COVID-19 cases, 78% had Danish origin, 3% Western origin, and 19% non-Western origin. Regarding regional origin, the majority came from Asia (10%) followed by Europe (9%) and Africa (2%). Large intergroup variations were shown for age on admission in that 84% of Danish origin were > 50 years whereas this was only the case for 65% of non-Western origin and 76% of Western origin. Distribution of sex among hospitalised was nearly identical in the three groups. Compared to individuals of Danish origin, Western and no-Western migrants had fewer comorbidities. More non-Western migrants had low educational level, lived in more dense neighbourhoods, or were categorised into the lowest income quartiles compared to the other two groups. Among all inpatients, 977 (15.3%) died and 342 (5.3%) received MV. Of those who died, 89% were of Danish origin, 3% of Western origin, and 8% of non-Western origin.

**Table 1:** Population characteristics of COVID-19 hospitalizations (n = 6406), deaths (n = 977) and mechanical ventilation (n = 342) based on Danish national data during

		Danish origin						Western origin*				Non-Western origin					
		Hospitalized COVID-19 cases, N = 4992		Deaths with COVID-19, N = 868		COVID-19 cases treated with MV, N = 249		Hospitalized COVID-19 cases, N = 225		Deaths with COVID-19, N = 30		Hospitalized COVID-19 cases, N = 1189		Deaths with COVID-19, N = 79		COVID-19 cases treated with MV, N = 84	
*Characteristics*		No.	%	No.	%	No.	%	No.	%	No.	%	No.	%	No.	%	No.	%
**Age, median (IQR)**	**Years**	73 (57–82)		83 (77–89)		70 (63–77)		65 (50–79)		85 (80–91)		57 (45–68)		77 (70–82)		63 (55–74)	
**Age groups**	**< 50 years**	777	16	4	1	12	5	55	24	0	0	411	35			13	15
	**≥ 50 years**	4215	84	864	99	237	95	170	76	30	100	778	65			71	84
**Sex**	**Women**	2321	47	369	42	82	33	107	48	14	47	534	45	23	29	19	23
**Migrant background**	**Immigrant**							209	93	25	83	1087	91	79	100		
	**Descendant**							16	7	5	17	102	9	0	0		
**Length of residence**	**≤ 5 years**							19	9	0	0	70	6				
	**6–15 years**							46	22	0	0	127	12				
	**16–29 years**							31	14	3	12	416	38				
	**30 + years**							113	54	22	88	474	44				
**Marital status**	**Unmarried**	2568	51	526	61	83	33	117	52	17	57	416	35	37	47	23	27
**Educational level**	**Low**	1501	30	370	44	80	32	44	22	11	44	479	46	40	46	42	55
	**Medium**	2092	42	349	41	118	48	78	38	5	20	325	31	12	31	19	25
	**High**	1324	27	124	15	49	20	80	40	9	36	249	23	9	23	15	20
**Charlson Comorbidity Index**	**0**	2759	56	294	34	130	52	140	62	9	30	795	67	31	39	53	63
	**1**	890	18	190	22	48	19	31	14	9	30	183	15	16	20	14	17
	**2**	622	12	141	16	41	17	28	12	5	17	105	9	13	16	9	11
	**≥ 3**	721	14	243	28	30	12	26	12	7	23	106	9	19	24	8	9
**No. of persons / household**	**1**	2006	40	553	64	71	28	89	39			206	17	27	34	8	10
	**2**	2068	41	285	33	149	60	62	27			309	26	29	37	32	38
	**3**	404	8	15	1	14	6	26	12			178	15	5	6	17	20
	**4**	331	7	6	1	9	4	24	11			199	17	3	4	13	15
	**≥ 5**	183	4	9	1	6	2	24	11			297	25	15	19	14	17
**Occupational status**	**Employed**	1716	35	25	3	84	34	110	49			433	37			24	29
	**Other inactivities**	3144	62	840	97	157	63	110	49			549	46			54	64
	**Unemployed**	132	3	3	0.4	8	3	5	2			206	17			6	7
**Income**	**1st quartile**	1050	21	257	30	32	13	56	25	8	27	681	57	58	73	49	58
	**2nd quartile**	1648	33	412	47	94	38	71	32	11	37	274	23	17	21	19	23
	**3rd quartile**	1030	21	131	15	56	22	51	22	6	20	147	12	4	5	13	15
	**4th quartile**	1264	25	68	8	67	27	47	21	5	17	87	7	0	0	3	4
**Population density**	**< 83 prs/km2**	1468	29	235	27	66	26	45	20			233	20	11	14	7	8
	**83–310 prs/km2**	1206	24	143	16	74	30	30	13			154	13	7	9	18	21
	**≥ 310 prs/km2**	2318	46	490	56	109	44	150	67			802	67	61	77	59	70
**World regional origin**	**Africa**											134	12	10	13	15	18
	**Asia**											651	56	38	49	46	55
	**Denmark**	4992	100	868	100	249	100										
	**Europe**							210	93	26	87	371	32	29	38	22	27

* There were too few COVID-19 cases to be presented in tables for individuals of Western origin where cells are empty or not there for mechanical ventilation

Table 2 shows crude and stepwise adjusted ORs. Immigrants (OR:0.55; 95%CI: 0.44–0.70) and individuals of non-Western origin (OR: 0.49; 95% CI: 0.37–0.65) had a lower odds of death upon admission with COVID-19 compared to individuals of Danish origin. Mortality of individuals of Western origin or descendants did not differ statistically significantly compared to individuals of Danish origin. Regarding regional origin, individuals originating from Europe (OR: 0.60; 95% CI: 0.44–0.82) and Asia (OR: 0.45; 95% CI: 0.31–0.66) had a significantly lower mortality compared to individuals of Danish origin, whereas no significant differences were found for individuals originating from Africa. Among COVID-19 cases from Asia, 56% were from Middle Eastern countries, while most cases of deaths originating from Africa were Moroccans (60%), and the majority of deaths from Europe were among Turks (48%). The odds of mortality was lower for immigrants and descendants compared to individuals of Danish origin regardless of age group. The adjusted OR for individuals 65 years or older was 0.68 (95% CI: 0.52–0.88) and 0.57 (95% CI: 0.25–0.88) for individuals younger than 65 years.

**Table 2:** Stepwise odds of 30-day mortality among hospitalized COVID-19 cases in Denmark by immigrant, descendants, Western and non-Western origin, and by regions of origin*

			Crude	+ Sex	+ Age	+ CCI	+ Household size	+ Income
**Group**	**COVID-19 cases** **N = 6406**	**Deaths** **N = 977**	**OR (95% CI)**	**OR (95% CI)**	**OR (95% CI)**	**OR (95% CI)**	**OR (95% CI)**	**OR (95% CI)**
**Danish origin (ref.)**	4992	868	1.00	1.00	1.00	1.00	1.00	1.00
**Immigrants**	1273	104	0.41 (0.33–0.50)	0.40 (0.32–0.49)	0.47 (0.38–0.58)	0.53 (0.42–0.65)	0.66 (0.52–0.83)	0.55 (0.44–0.70)
**Descendants**	118	5	0.20 (0.08–0.50)	0.20 (0.08–0.50)	2.08 (0.71–6.12)	2.07 (0.68–6.29)	2.23 (0.67–7.40)	1.95 (0.59–6.48)
**Danish origin (ref.)**	4992	868	1.00	1.00	1.00	1.00	1.00	1.00
**Western origin**	225	30	0.73 (0.49–1.08)	0.73 (0.50–1.08)	0.83 (0.55–1.23)	0.85 (0.56–1.27)	0.81 (0.53–1.22)	0.78 (0.51–1.19)
**Non-Western origin**	1189	79	0.34 (0.27–0.43)	0.34 (0.27–0.43)	0.43 (0.34–0.55)	0.46 (0.36–0.59)	0.61 (0.47–0.80)	0.49 (0.37–0.65)
**Denmark (ref.)**	4992	868	1.00	1.00	1.00	1.00	1.00	1.00
**Africa**	134	10	0.38 (0.20–0.73)	0.38 (0.20–0.72)	0.50 (0.26–0.96)	0.55 (0.28–1.08)	0.70 (0.35–1.39)	0.55 (0.28–1.10)
**Asia**	651	38	0.30 (0.21–0.41)	0.29 (0.21–0.41)	0.39 (0.27–0.54)	0.42 (0.30–0.59)	0.55 (0.39–0.80)	0.45 (0.31–0.66)
**Europe**	581	55	0.50 (0.37–0.66)	0.50 (0.37–0.66)	0.58 (0.43–0.78)	0.60 (0.45–0.81)	0.70 (0.51–0.95)	0.60 (0.44–0.82)

OR: Odds ratio; CI: confidence intervals

* The analyses of region of origin were based on 6358 individuals. Most deaths among COVID-19 cases from Africa were from Morocco (62%) and Somalia (37%), while the majority of deaths from Asia originated from Middle Eastern countries (56%) including Pakistan (32%), and Afghanistan (12%), and most deaths among the cases from Europe were from Turkey (48%), East European countries (35%), and Germany (17%)

Table 3 shows that immigrants and descendants (OR: 1.62; 95% CI: 1.22–2.15) and individuals of non-Western origin (OR: 1.83; 95% CI: 1.35–2.47) had a significantly higher odds of receiving MV during admission with COVID-19 compared to Danish born individuals, whereas individuals with Western origin did not differ significantly. Regarding region of origin, individuals originating from Asia (OR: 1.92; 95% CI: 1.33–2.78) and Africa (: 3.07; 95% CI: 1.70–5.53) had a higher odds of receiving MV, whereas no differences were found for individuals from Europe compared to individuals of Danish origin.

**Table 3:** Stepwise odds of mechanical ventilation among hospitalized COVID-19 cases in Denmark by immigrant/descendants, Western and non-Western origin, and by regions of origin*

			Crude	Sex	+ Age	+ CCI	+ Household size	+ Income
**Group**	**COVID-19 cases**	**MV**	**OR (95%CI)**	**OR (95%CI)**	**OR (95%CI)**	**OR (95%CI)**	**OR (95%CI)**	**OR (95%CI)**
**Danish origin (ref.)**	5046	249	1.00	1.00	1.00	1.00	1.00	1.00
Immigrants and descendants**	1443	93	1.33 (1.04–1.70)	1.32 (1.03–1.68)	1.52 (1.19–1.96)	1.51 (1.18–1.94)	1.56 (1.19–2.03)	1.62 (1.22–2.15)
**Danish origin (ref.)**	5046	249	1.00	1.00	1.00	1.00	1.00	1.00
**Western origin**	228	9	0.79 (0.40–1.56)	0.80 (0.41–1.58)	0.86 (0.44–1.70)	0.85 (0.43–1.69)	0.92 (0.47–1.83)	0.92 (0.46–1.82)
**Non-Western origin**	1215	84	1.43 (1.11–1.85)	1.41 (1.09–1.83)	1.66 (1.28–2.16)	1.65 (1.27–2.14)	1.70 (1.28–2.25)	1.83 (1.35–2.47)
**Denmark (ref.)**	5046	249	1.00	1.00	1.00	1.00	1.00	1.00
**Africa**	138	15	2.35 (1.36–4.08)	2.26 (1.30–3.92)	2.66 (1.52–4.67)	2.61 (1.49–4.59)	2.76 (1.55–4.90)	3.07 (1.70–5.53)
**Asia**	665	46	1.43 (1.03–1.98)	1.41 (1.02–1.96)	1.70 (1.22–2.36)	1.68 (1.20–2.34)	1.76 (1.24–2.50)	1.92 (1.33–2.78)
**Europe**	592	31	1.07 (0.73–1.56)	1.07 (0.73–1.58)	1.19 (0.81–1.75)	1.18 (0.80–1.74)	1.22 (0.82–1.80)	1.25 (0.84–1.87)

OR: Odds ratio; CI: confidence intervals

* The analyses of region of origin were based on 6441 individuals. Most deaths among COVID-19 cases from Africa were from Morocco (40%) and Somalia (60%), while the majority of dead cases from Asia originated from Middle Eastern countries (58%) Pakistan (32%), and Afghanistan (10%), and most deaths among the cases from Europe were from Turkey (52%) and East European countries (48%)

**Immigrants and descendants are combined in this analyses as numbers otherwise would be insufficient

Table 4 shows differences in odds of death among COVID-19 patients treated with MV. No significant differences were found between immigrants and descendants nor individuals of non-Western or Western origin compared to individuals of Danish origin. Likewise, for world regional origin, there were no differences found among the different regional groups compared to Danish born individuals.

**Table 4:** Stepwise odds of death among COVID-19 patients treated with mechanical ventilation grouped by immigrant/descendants, Non-Western origin, and regions of origin. Follow-up period from 03.03.2020–02.02.2021*

			Crude	Sex	+ Age	+ CCI	+ Household size	+ Income
**Group**	**COVID-19 cases** N = 342	**Deaths** N = 149	**OR (95%CI)**	**OR (95%CI)**	**OR (95%CI)**	**OR (95%CI)**	**OR (95%CI)**	**OR (95%CI)**
**Danish origin (ref.)**	249	109	1.00	1.00	1.00	1.00	1.00	1.00
Immigrants and descendants**	93	40	0.97 (0.60–1.57)	0.94 (0.58–1.52)	0.98 (0.60–1.60)	1.07 (0.64–1.77)	0.91 (0.52–1.59)	0.75 (0.39–1.45)
**Danish origin (ref.)**	249	109	1.00	1.00	1.00	1.00	1.00	1.00
**Non-Western origin**	84	37	1.01 (0.61–1.66)	0.98 (0.59–1.62)	1.03 (0.62–1.73)	1.10 (0.65–1.87)	0.97 (0.54–1.74)	0.78 (0.39–1.57)
**Denmark (ref.)**	249	109	1.00	1.00	1.00	1.00	1.00	1.00
**Africa**	15	6	0.86 (0.30–2.48)	0.79 (0.27–2.31)	0.79 (0.27–2.29)	0.85 (0.29–2.52)	0.86 (0.27–2.73)	0.68 (0.19–2.42)
**Asia**	46	18	0.83 (0.43–1.57)	0.80 (0.42–1.52)	0.86 (0.44–1.70)	0.95 (0.47–1.90)	0.78 (0.37–1.63)	0.60 (0.26–1.36)
**Europe**	31	15	1.20 (0.57–2.54)	1.20 (0.57–2.55)	1.19 (0.56–2.51)	1.33 (0.61–1.90)	1.06 (0.45–2.47)	0.98 (0.39–2.45)

OR: Odds ratio; CI: confidence intervals

* Individuals of Western origin were excluded from these analyses due to few cases. The analyses of individuals of Danish and Western origin and world regions were based on 333 and 341 individuals respectively. Most deaths among COVID-19 cases from Africa were from Morocco and Somalia, while the majority of dead cases from Asia originated from Middle Eastern countries (41%) Pakistan (41%), and Afghanistan (18%), and most deaths among the cases from Europe were from Turkey (46%) and East European countries (54%)

**Immigrants and descendants are combined in this analyses as numbers otherwise would be insufficient

## Discussion

In this unique nationwide register-based study of ethnic disparities in 30-day mortality and use of MV during the first year of the pandemic, we highlight several findings. First, immigrants and individuals of non-Western origin had a significantly lower mortality compared to individuals of Danish origin. Second, immigrants, descendants, individuals of non-Western origin and individuals originating from Asia had a significantly higher risk of receiving MV compared to individuals of Danish origin. No significant intergroup differences were found in mortality among those who had received with mechanical ventilation.

First, our results showed that albeit immigrants of non-western origin had a higher rate of admission from COVID-19 in Denmark [9], they did not have a higher risk of 30-day mortality. Studies from the United States and the United Kingdom showed a higher population-level mortality among ethnic minorities infected with COVID-19 at a population level [3, 10, 12]. Our study analysed hospital-level mortality and this likely explains the discrepancy. Our results are in line with a meta-analysis based on 58 studies concluding that the risk of COVID-19 was higher in most ethnic minorities, but once hospitalised, there were no differences in COVID-19 outcomes [14]. The European studies included in the metaanalysis were from the UK, Italy, and Spain; and included diverse ethnic/racial groups. A more recent Spanish study was similarly unable to show a difference in COVID-19 mortality between hospitalised Europeans and non-Europeans [22]. Our results may be explained by a lower threshold (less severe disease) for hospitalising immigrants with non-Western background due to well-known barriers related to communication and health literacy resulting in uncertainties in the clinical assessment [23, 24]. There were marked differences in age upon admission with immigrants being markedly younger, but age was accounted for in our analyses. Comorbidity could also play a role in immigrants’ lower mortality, as younger people are less likely to have comorbidities. However, adjustment for covariates including comorbidity and sociodemographic factors did not change any of our results markedly. Not all diseases related to the prognosis of COVID-19 are included in the CCI score, i.e. asthma, as it was developed for other purposes, however, the CCI score has been validated as a prognostic marker of severe COVID-19 disease and death [25]. It should be mentioned that descendants showed somewhat contrasting results, but the sample size was very small, which warrants further follow-up for example in combined cross-country data sets.

Second, we found that immigrants of non-Western origin were more likely to receive MV than individuals of Danish origin. Here we were not able to divide immigrants and descendants. The higher use of MV among immigrants is in line with previous findings [14, 22]. Again, we did not see any group differences in mortality among those, who received MV indicating equity in quality of intensive care including referral and treatment. It is not straightforward to explain the contrast between lower mortality and higher risk of receiving mechanical ventilation among migrants of non-Western origin in our study. Unfortunately, we were unable to include clinical indicators of disease severity during admission such as oxygen need and biomarkers. Future studies should include disease severity markers in order to account for possible difference at presentation. In addition, immunological and genetic factors have not been taken into account. Finally, communication barriers regarding optimal use of oxygen and ability to cooperate across language barriers with limited quality in access to interpreters due high flow oxygen treatment; and limited visits by families due to restrictions could possibly result in worse disease severity during admission and a higher risk of MV.

Our study has several methodological strengths. First, it is based on unique nationwide data representing all hospitalised COVID-19 cases in Denmark from March 2020 to February 2021. Use of national data made it possible to include the entire population and link with different national registers including a number of relevant confounders on an individual level. Second, the validity of COVID-19 diagnoses is increased by only including cases where COVID-19 was the primary reason for hospital contact. Third, we were able to divide migrants into several subcategories based on country of origin. The study also has several limitations. First, on a positive note Denmark’s management of the pandemic through restrictive policies resulted in a comparatively limited number of hospitalisations restricting the sample size of specific country groups and reducing statistical power in some analyses. Second, the study population only included the most severe COVID-19 cases hospitalized for ≥ 24 h and is not representative of COVID-19 cases in the general population. Therefore, our results are not generalizable to mortality from COVID-19 in the general population.

## Conclusion

Our study showed that among individuals, who had been admitted for COVID-19, immigrants as well as individuals of non-Western origin had a significantly lower mortality compared to individuals of Danish origin, however, they had a significantly higher risk of MV. No significant differences between any of the groups were found in mortality among those with MV. To disentangle these complex relationships, future research on mortality and MV should ideally include clinical and biomarker data as well as qualitative data related to interpreter use and patients’ experiences of cultural competency among health care professionals.
